# Recent Insights Into Electronic Performance, Magnetism and Exchange Splittings in the Cr-substituted CaO

**DOI:** 10.3389/fchem.2020.00526

**Published:** 2020-07-10

**Authors:** Bendouma Doumi, Allel Mokaddem, Abdelkader Tadjer, Adlane Sayede

**Affiliations:** ^1^Department of Physics, Faculty of Sciences, Dr. Tahar Moulay University of Saida, Saida, Algeria; ^2^Instrumentation and Advanced Materials Laboratory, University Center of Nour Bachir El-Bayadh, El-Bayadh, Algeria; ^3^Technology Department, University Center of Nour Bachir El-Bayadh, El-Bayadh, Algeria; ^4^Modelling and Simulation in Materials Science Laboratory, Physics Department, Djillali Liabes University of Sidi Bel-Abbes, Sidi Bel-Abbes, Algeria; ^5^University of Artois, CNRS, Centrale Lille, Univ. Lille, UMR 8181 – UCCS – Unité de Catalyse et Chimie du Solide, Lens, France

**Keywords:** first-principles calculations, electronic structures, ferromagnetism, exchange splittings, spintronics

## Abstract

The first-principles computations of density functional theory are employed to characterize the structural properties, electronic structures, and ferromagnetism induced by Cr impurities in Ca_1-x_Cr_x_O compounds at concentrations *x* = 0. 25, 0.5, and 0.75. The dynamic stability is performed by the phonon spectra calculations. The structural parameters are computed by using Wu-Cohen generalized gradient approximation, while the electronic and magnetic properties are determined by the accurate Tran–Blaha-modified Becke–Johnson exchange potential. The crystal field, direct and indirect exchange splittings were investigated to determine the origin and stability of ferromagnetic state configuration. The Ca_1-x_Cr_x_O systems have right half-metallicities, which are verified by the spin polarization of 100% and the integer values of total magnetic moments. The Ca_0.75_Cr_0.25_O, Ca_0.5_Cr_0.5_O, and Ca_0.25_Cr_0.75_O are half-metallic ferromagnetic with flip-gaps of 1.495, 0.888, and 0.218 eV, respectively. Therefore, the Ca_1-x_Cr_x_O materials are suitable candidates for possible applications of spin-injection in future semiconductors spintronics.

## Introduction

In recent years, the exploitation of new materials for technological development has become an attractive area of research for the scientific community. Spintronics is a modern field emerging from quantum electronics relating to exploration and control of the electron spin; it is currently an important research topic because the spin-based devices have interesting multifunctional characteristics such as non-volatility, data-processing speed, higher integration densities (Elilarassi and Chandrasekaran, 2017; Rai et al., 2018). In the last years, the half-metal ferromagnetic (HMF) materials have attracted considerable interest due to their wide application in spintronic devices as magnetic sensors, tunnel junctions, and spin injection sources of electrons (Özdemir and Merdan, 2019). The diluted magnetic semiconductors (DMSs) are obtained from the III–V, II–VI, or IV–VI semiconductors doped with magnetic elements, inducing changes in their structural, electronic, and magnetic properties, which are related to the concentration of magnetic impurities. These materials have received a lot of attention for scientific research because they are promising candidates for innovative applications in spintronics, as an example for non-volatile memories, optical switches, and magneto-optical devices (Ohno, 1998; Dietl et al., 2000; Wolf et al., 2001; Zuti et al., 2004; Sato et al., 2010; Kumar et al., 2014).

Alkaline earth chalcogenides are important materials with a wide technological application ranging from catalysis to microelectronics as well as in the area of electro- and photo-luminescent devices (Hakamata et al., 2005; Speziale et al., 2006; Murtaza et al., 2014). Among these compounds, the CaX (X = O, S, Se) has a closed-shell ionic environment, crystallizing in the rock-salt NaCl-type (B1) structure at ambient conditions (Doll et al., 1996; Mishra et al., 2012). Under high pressure, they undergo a first-order structural phase transition from the NaCl-type (B1) to the CsCl-type (B2) crystal structure (Luo et al., 1994; Doll et al., 1996; Dadsetani and Doosti, 2009; Mishra et al., 2012). The CaO calcium oxide can be a potential candidate as a DMS material according to several theoretical studies (Kenmochi et al., 2004; Kemmochi et al., 2005; An Dinh et al., 2006). Kenmochi et al. (2004) have studied a new class of DMSs based on CaO without transition metals, where the substituted CaO by C and N at the oxygen sites shows room-temperature ferromagnetism with half-metallic property. The ferromagnetism in C-doped CaO is explained by the exchange coupling constants, where it is induced by the strong hybridization between 2p states of carbon and 2p states of oxygen (An Dinh et al., 2006). Further, Kemmochi et al. (2005) have found that the stability of ferromagnetic state in the C-doped CaO is originated from the Zener's double-exchange mechanism.

In this study, we have investigated the structural parameters, electronic performance, magnetism, and exchange splittings of Ca_1-x_Cr_x_O materials at various concentrations *x* = 0, 0.25, 0.5, and 0.75. The calculations are performed by the use of first-principle approaches of density functional theory (DFT) (Hohenberg and Kohn, 1964; Kohn and Sham, 1965) as implemented in the WIEN2k package (Blaha et al., 2001). The phonon spectra are used to verify the dynamic stability of Ca_1-x_Cr_x_O materials. The exchange and correlation potential is evaluated by the generalized gradient approximation of Wu and Cohen (GGA-WC) (Wu and Cohen, 2006), and the Tran–Blaha-modified Becke–Johnson potential (TB-mBJ) (Tran and Blaha, 2009).

## Computational Methods

The calculations of structural, electronic, and magnetic properties of Ca_1-x_Cr_x_O are carried out using the WIEN2k code (Blaha et al., 2001) based on the first-principle computations of DFT and the full-potential linearized augmented plane-wave (FP-LAPW) method (Singh and Nordstrom, 2006). We have computed the structural parameters using GGA-WC approximation (Wu and Cohen, 2006), while the electronic and magnetic properties are determined by employing the TB-mBJ exchange potential (Becke and Johnson, 2006; Tran and Blaha, 2009). The phonon frequencies are determined by the phonopy code (Togo and Tanaka, 2015) based on the density functional perturbation theory (Giannozzi et al., 1991; Gonze and Lee, 1997). The phonon spectra are calculated using the WIEN2k and the phonopy packages to verify the dynamic stability of Ca_1-x_Cr_x_O doping compounds.

The averages of Muffin-tin spheres radii are selected for Ca, Cr, and O atoms in condition that Muffin-tin spheres do not overlap. The cutoff of −6 Ry is utilized to separate between the valence and core states. The basic functions and the potentials are extended in combination of spherical harmonics around the atomic sites, that is to say, the atomic spheres with a cutoff *l*_max_ = 10, and in the interstitial region are extended into plane waves with a cutoff *R*_MT_*k*_max_ = 8 (where *R*_MT_ is the average radius of the Muffin-tin spheres). We have expanded the charge density in Fourier up to *G*_max_ = 14 (a.u.)^−1^, where *G*_max_ represents the largest vector in the Fourier expansion. The calculations were performed for structures of eight (8) atoms of Ca_1-x_Cr_x_O at concentrations *x* = 0, 0.25, 0.5, and 0.75 such as CaO, Ca_0.75_Cr_0.25_O, Ca_0.5_Cr_0.5_O, and Ca_0.25_Cr_0.75_O supercells. The integration of the Brillouin zone was carried out by the Monkhorst–Pack mesh (Monkhorst and Pack, 1976) using the special k-points of (10 × 10 × 10) for CaO and (5 × 5 × 5) for Ca_0.75_Cr_0.25_O, Ca_0.5_Cr_0.5_O, and Ca_0.25_Cr_0.75_O compounds. The self-consistent is achieved when the total energy convergence condition was set to be 10^−1^ mRy.

## Results and Discussion

### Structural Properties

#### Crystal Structures

The calcium oxide CaO is known as a wide-band gap II–VI semiconductor, crystallizing in rock-salt NaCl (B1) structure at ambient conditions (Doll et al., 1996; Mishra et al., 2012). The primitive unit cell of CaO contains two atoms such as the calcium (Ca) and the oxygen (O), which belong, respectively, to the main groups II and VI of the periodic table. Our study is based on the conventional NaCl structure of eight atoms of CaO such as Ca_4_O_4_, where the Ca is located at (0, 0, 0) position and the O atom at (0.5, 0.5, 0.5) with space group of $Fm\bar{3}m$ No. 225. The Ca_3_CrO_4_, Ca_2_Cr_2_O_4_, and CaCr_3_O_4_ supercells are created by substituting one, two, and three Cr impurities at Ca sites, respectively. Consequently, we have obtained three supercells such as Ca_0.75_Cr_0.25_O at concentration *x* = 0.25, Ca_0.5_Cr_0.5_O at *x* = 0.5 and Ca_0.25_Cr_0.75_O at *x* = 0.75. The Ca_0.75_Cr_0.25_O and Ca_0.25_Cr_0.75_O have cubic lattices with space group of $Pm\bar{3}m$ No 221, while the Ca_0.5_Cr_0.5_O supercell has a tetragonal lattice with space group of *P4/mmm* No. 123. We have noticed that all symmetry operations have been considered in the initialization of structures of Ca_1-x_Cr_x_O supercells for computations of different properties.

#### Structural Stability

The structural stability of the Ca_1-x_Cr_x_O compounds in the ferromagnetic rock-salt NaCl (B1) structure is verified by computing the formation energies. The formation energies (*E*_form_) of the Ca_4–y_Cr_*y*_O_4_ materials are determined using the following expression (Bai et al., 2011; Doumi et al., 2015a):

(1) $$\begin{array}{l} E_{\text{form}}=E_{\text{total}}\left(\text{C}{\text{a}}_{4-y}\text{C}{\text{r}}_{y}{\text{O}}_{4}\right)-\left(\frac{\left(4-y\right)\;E\left(\text{Ca}\right)}{8}\right)-\left(\frac{y\;E\left(\text{Cr}\right)}{8}\right)\\ \;-\left(\frac{4\;E\left(\text{O}\right)}{8}\right) \end{array}$$

where *E*_total_(Ca_4–y_Cr_*y*_O_4_) corresponds to the minimum total energy of Ca_4–y_Cr_*y*_O_4_ per atom, and *E*(Ca), *E*(Cr), and *E*(O) refer to the minimum total energies per atom of bulks Ca, Cr, and O, respectively. The *y* = 1, 2, and 3 are the number of substituted Cr impurities in Ca_4–y_Cr_*y*_O_4_ supercells. We have found that the formation energies are −5.99, −6.37, and −6.76 eV, respectively, for Ca_0.75_Cr_0.25_O, Ca_0.5_Cr_0.5_O, and Ca_0.25_Cr_0.75_O. Therefore, the negative values of formation energies suggest that these compounds are thermodynamically stable in the ferromagnetic rock-salt structure.

**Figure 1:**
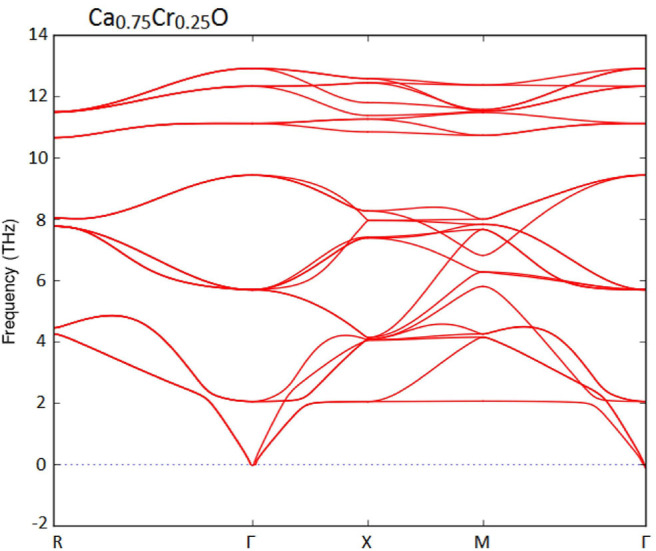
Phonon spectra for Ca_0.75_Cr_0.25_O.

**Figure 2:**
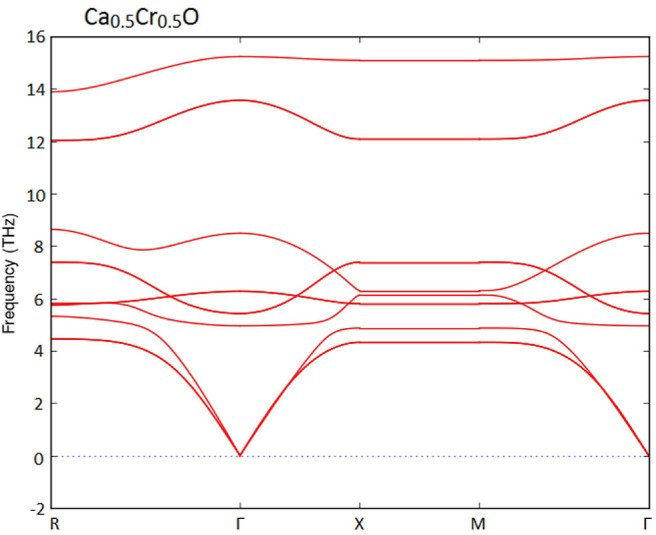
Phonon spectra for Ca_0.5_Cr_0.5_O.

**Figure 3:**
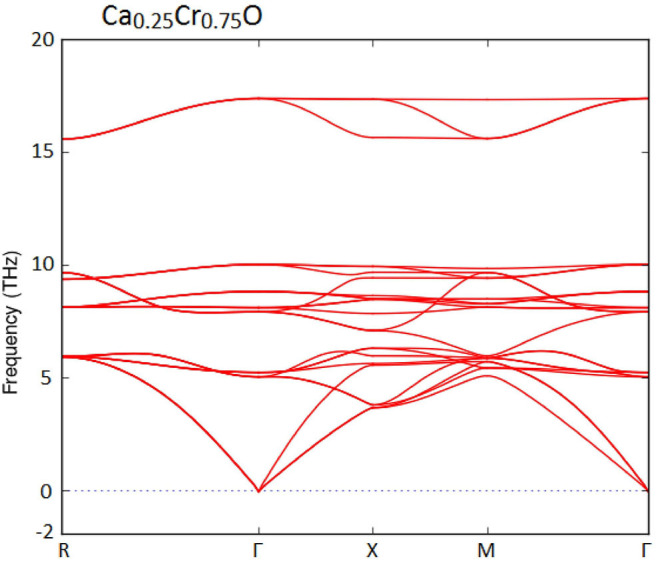
Phonon spectra for Ca_0.25_Cr_0.75_O.

Furthermore, we have used the phonon spectra to demonstrate the dynamic stability of the Ca_1-x_Cr_x_O doping compounds. The diagrams of phonon frequencies at high symmetry points in the Brillouin zone of Ca_0.75_Cr_0.25_O, Ca_0.5_Cr_0.5_O, and Ca_0.25_Cr_0.75_O are shown by Figures 1–3, respectively. These figures depicted that all branches of phonons have positive frequencies, and there are no negative frequencies (Deng et al., 2019), confirming the dynamic stability of Ca_0.75_Cr_0.25_O, Ca_0.5_Cr_0.5_O, and Ca_0.25_Cr_0.75_O compounds.

#### Equilibrium Structural Parameters

The variations of total energies as a function of volumes of Ca_1-x_Cr_x_O compounds are fitted by Murnaghan (1944) equation of state to determine the equilibrium structural parameters. Table 1 summarizes the lattice constants, the bulk modules, and their first derivatives with other experimental data (Mammone et al., 1981; Kaneko et al., 1982; Richet et al., 1988) and theoretical values (Tran et al., 2007; Wu et al., 2014; Cinthia et al., 2015; Fan et al., 2015; Nejatipour and Dadsetani, 2015; Santana et al., 2016; Yang et al., 2016; Salam, 2018) computed by the generalized gradient approximation of Perdew–Burke–Ernzerhof (GGA-PBE) (Perdew et al., 1996), revised GGA of Perdew–Burke–Ernzerhof (GGA-PBEsol) (Perdew et al., 2008) and the local density approximation (LDA) (Perdew and Zunger, 1981; Perdew and Wang, 1992).

**Table 1:** Computed structural parameters such as lattice constants (*a*), bulk modules (*B*), and their pressure derivatives (*B*′) for Ca_1-x_Cr_x_O materials at concentrations *x* = 0, 0.25, 0.5, and 0.75 with other experimental and theoretical data.

**Material**	***a* (Å)**	***B* (GPa)**	***B^**′**^***	**Method**	**References**
This work				Generalized gradient approximation of Wu and Cohen (GGA-WC)	
CaO	4.772	113.15	4.44		
Ca_0.75_Cr_0.25_O	4.660	126.40	4.63		
Ca_0.5_Cr_0.5_O	4.549	128.91	3.63		
Ca_0.25_Cr_0.75_O	4.415	133.23	3.75		
Other calculations					
CaO	4.812			Experimental	Kaneko et al., 1982
		111	4.2	Experimental	Richet et al., 1988
	4.8105	115	4.1	Experimental	Mammone et al., 1981
	4.777	116		GGA-WC	Tran et al., 2007
	4.77			GGA-WC	Fan et al., 2015
	4.841	105		Generalized gradient approximation of Perdew–Burke–Ernzerhof (GGA-PBE)	Tran et al., 2007
	4.81			GGA-PBE	Fan et al., 2015
	4.857			GGA-PBE	Wu et al., 2014
	4.843	107	4.2	GGA-PBE	Cinthia et al., 2015
	4.84			GGA-PBE	Nejatipour and Dadsetani, 2015
	4.834			GGA-PBE	Yang et al., 2016
	4.836	102.3	4.17	GGA-PBE	Salam, 2018
	4.77			Revised GGA of Perdew–Burke–Ernzerhof (GGA-PBEsol)	Fan et al., 2015
	4.719	129		Local density approximation (LDA)	Tran et al., 2007
	4.71			LDA	Fan et al., 2015
	4.734	119.54	4.23	LDA	Santana et al., 2016

The results of lattice constants and bulk modulus of CaO are closed to theoretical values of Tran et al. (2007) and Fan et al. (2015) calculated with GGA-WC potential (Wu and Cohen, 2006). We see that there is a good agreement between our calculations and experimental results (Mammone et al., 1981; Kaneko et al., 1982; Richet et al., 1988) and theoretical value found by GGA-PBE sol (Perdew et al., 2008). On the other hand, the obtained values of these parameters with GGA-WC (Wu and Cohen, 2006) are better than the calculations of Tran et al. (2007), Wu et al. (2014) Cinthia et al. (2015), Nejatipour and Dadsetani (2015), Yang et al. (2016), Salam (2018), Tran et al. (2007), Fan et al. (2015), and Santana et al. (2016) found, respectively, by GGA-PBE (Perdew et al., 1996) and LDA approximation (Perdew and Zunger, 1981; Perdew and Wang, 1992). The performance of GGA-WC approximation for predicting structural parameters is due to the fourth-order gradient expansion of exchange-correlation functional (Doumi et al., 2015b; Sajjad et al., 2015). For the Ca_1-x_Cr_x_O doping compounds, the ionic radius of Cr is lower than that of Ca atom, leading to decrease in the lattice constant with increasing Cr concentration. Consequently, the bulk modulus of Ca_1-x_Cr_x_O increases with increasing concentration of Cr. The Ca_1-x_Cr_x_O doping compound becomes harder as the concentration of Cr increases.

### Electronic Properties

#### Band Structures and Half-Metallicity

**Figure 4:**
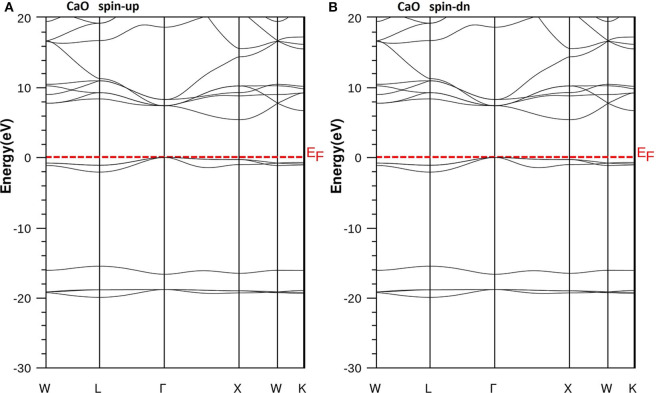
Band structures of CaO. **(A)** Majority spins (*up*) and **(B)** minority spins (*dn*). The Fermi level is set to zero (*horizontal dotted line*).

**Figure 5:**
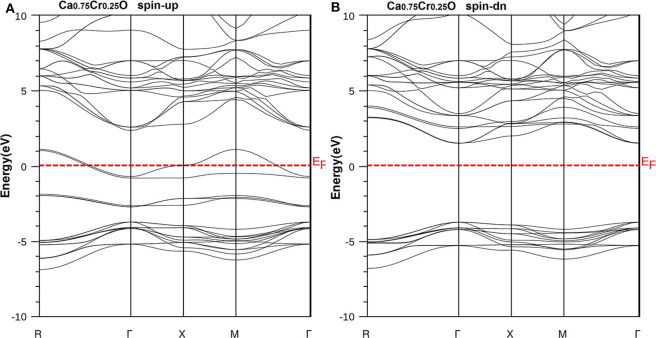
Band structures of Ca_0.75_Cr_0.25_O. **(A)** Majority spins (*up*) and **(B)** minority spins (*dn*). The Fermi level is set to zero (*horizontal dotted line*).

**Figure 6:**
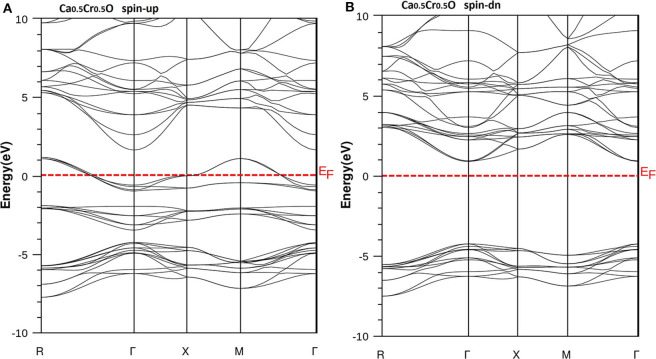
Band structures of Ca_0.5_Cr_0.5_O. **(A)** Majority spins (*up*) and **(B)** minority spins (*dn*). The Fermi level is set to zero (*horizontal dotted line*).

**Figure 7:**
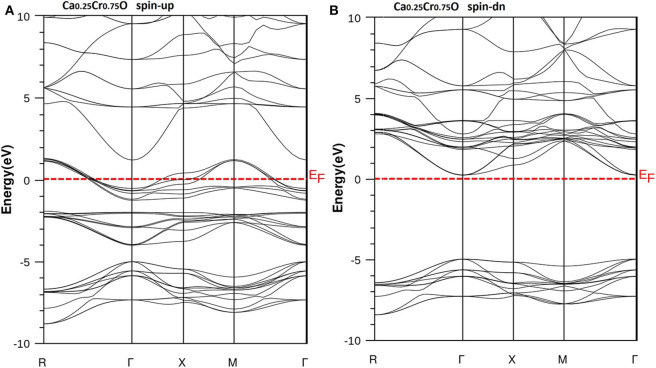
Band structures of Ca_0.25_Cr_0.75_O. **(A)** Majority spins (*up*) and **(B)** minority spins (*dn*). The Fermi level is set to zero (*horizontal dotted line*).

We have used the predicted structural parameters to characterize the electronic structures and the half-metallicity in Ca_1-x_Cr_x_O compounds. The TB-mBJ potential is used to determine the electronic structures and accurate band gaps of Ca_1-x_Cr_x_O compounds at concentration *x* = 0, 0.25, 0.5, and 0.75. The band structures of CaO, Ca_0.75_Cr_0.25_O, Ca_0.5_Cr_0.5_O, and Ca_0.25_Cr_0.75_O are shown, respectively, by the Figures 4–7.

According to Figure 4, the CaO is a semiconductor because both majority and minority spins revealed the analogous band structures with indirect gaps. The CaO has an indirect gap(*E*^Γ*X*^) between the valence bands maximum and conduction bands minimum situated, respectively, at Γ and X high symmetry points. Figures 5–7 demonstrate that the Ca_1-x_Cr_x_O for all concentrations are metallic and semiconductors, respectively, for majority- and minority-spin bands. Consequently, the Ca_1-x_Cr_x_O doping materials are half-metallic ferromagnets. The metallic character is due to localized partially filled 3d-Cr states of majority spins around Fermi level (*E*_F_), which interact with carriers of valence states of host semiconductor. For minority spins, the Ca_1-x_Cr_x_O doping systems keep a semiconductor behavior as CaO compound because the 3d-Cr empty minority-spin states arise in the bottom of conduction band minimum at Γ high symmetry point far than *E*_F_. Thus, the gap nature shifted from indirect band gap for CaO to direct gap for Ca_1-x_Cr_x_O doping systems.

The minority spins of Ca_1-x_Cr_x_O systems are characterized by direct wide-band gaps called half-metallic ferromagnetic (HMF) gaps located at the Γ high symmetry point. The minimal energies that separate the Fermi level (zero eV), and the conduction band minimums determine the half-metallic (HM) gaps. Table 2 shows the computed band gaps such as the direct HMF gaps (*E*_HMF_) and HM gaps (*E*_HM_) of Ca_0.75_Cr_0.25_O, Ca_0.5_Cr_0.5_O, and Ca_0.25_Cr_0.75_O, and the indirect gap (*E*^Γ*X*^) of CaO with other theoretical (Fan et al., 2015; Nejatipour and Dadsetani, 2015; Yang et al., 2016; Tran and Blaha, 2017; Salam, 2018) and experimental data (Whited et al., 1973). The result of wide-indirect gap of 5.392 eV of CaO is in good agreement with recent calculated value of 5.35 eV of Tran and Blaha (2017) study using the same TB-mBJ potential (Becke and Johnson, 2006; Tran and Blaha, 2009), and it is better than the values ranging from 3.437 to 3.67 eV of Fan et al. (2015), Nejatipour and Dadsetani (2015), Yang et al. (2016), Tran and Blaha (2017), Salam (2018) found by the LDA (Perdew and Zunger, 1981; Perdew and Wang, 1992), GGA-WC (Wu and Cohen, 2006), GGA-PBE (Perdew et al., 1996), and GGA-PBEsol exchange potentials (Perdew et al., 2008). The TB-mBJ provides accurate gap for CaO because he is known for its performance in the calculation of electronic structures of insulators and semiconductors with respect to LDA and all forms of GGA approximations (Bhattacharjee and Chattopadhyaya, 2017a,b; Chattopadhyaya and Bhattacharjee, 2017; Berriah et al., 2018).

**Table 2:** Computed band gaps such as indirect band gap (*E*^Γ*x*^) for CaO, half-metallic ferromagnetic (HMF) gaps (*E*_HMF_), and half-metallic (HM) gaps (*E*_HM_) of minority-spin bands for Ca_1-x_Cr_x_O at concentrations *x* = 0.25, 0.5, and 0.75 with other theoretical and experimental data.

**Material**	***E*_HMF_ (eV)**	***E*_HM_ (eV)**	**E^ΓX^ (eV)**	**Method**	
**This work**
CaO			5.392	Tran–Blaha-modified Becke–Johnson potential (TB-mBJ)	
Ca_0.75_Cr_0.25_O	5.242	1.495		TB-mBJ	
Ca_0.5_Cr_0.5_O	5.142	0.888		TB-mBJ	
Ca_0.25_Cr_0.75_O	5.195	0.218		TB-mBJ	
**Other calculations**					
CaO			3.53	GGA-WC	Fan et al., 2015
			3.647	GGA-PBE	Fan et al., 2015
			3.669	GGA-PBE	Nejatipour and Dadsetani, 2015
			3.65	GGA-PBE	Yang et al., 2016
			3.658	GGA-PBE	Salam, 2018
			3.67	GGA-PBE	Tran and Blaha, 2017
			3.518	GGA-PBEsol	Fan et al., 2015
			3.437	LDA	Fan et al., 2015
			3.49	LDA	Tran and Blaha, 2017
			5.35	TB-mBJ	Tran and Blaha, 2017
			7.00	Experimental	Whited et al., 1973

Moreover, the half-metallic gap or flip-gap is an interesting performance for exploring the spin-injection for a half-metallic material in spintronics. The Ca_0.75_Cr_0.25_O, Ca_0.5_Cr_0.5_O, and Ca_0.25_Cr_0.75_O compounds have the HM gaps of 1.495, 0.888, and 0.218 eV, respectively, situated between the conduction bands minimum (CBM) and *E*_F_. The CBM moves toward *E*_F_ due to widening of 3d-Cr empty states at the bottom of conduction band, and hence, the HM gap decreases from Ca_0.75_Cr_0.25_O, Ca_0.5_Cr_0.5_O, to Ca_0.25_Cr_0.75_O. The HM gap behavior is maintained for different concentrations, meaning that the Ca_1-x_Cr_x_O doping systems are right half-metallic materials.

#### Densities of States and Spin Polarization

**Figure 8:**
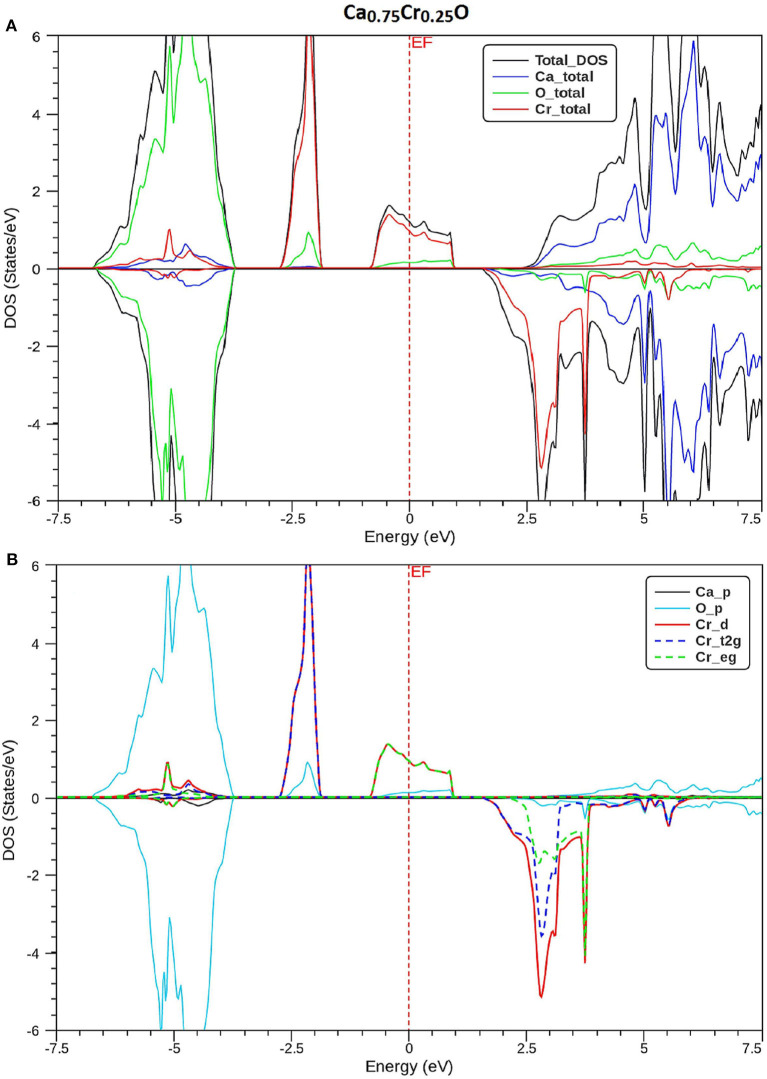
Spin-polarized total and partial densities of states (DOS) of Ca_0.75_Cr_0.25_O. The Fermi level is set to zero (*vertical dotted line*). **(A)** Total DOS of Ca, Cr, and O and **(B)** Partial DOS of p-Ca, p-O, d-Cr, t2g_d-Cr, and eg_d-Cr.

**Figure 9:**
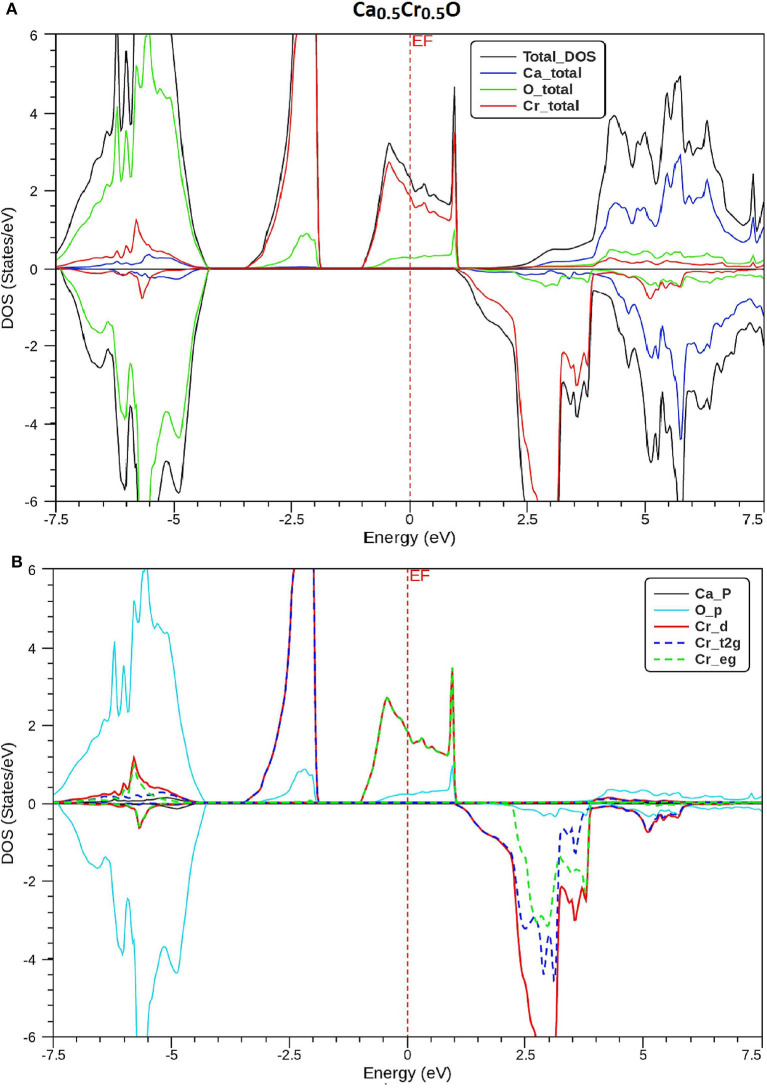
Spin-polarized total and partial densities of states (DOS) of Ca_0.5_Cr_0.5_O. The Fermi level is set to zero (*vertical dotted line*). **(A)** Total DOS of Ca, Cr, and O and **(B)** DOS of p-Ca, p-O, d-Cr, t2g_d-Cr, and eg_d-Cr.

**Figure 10:**
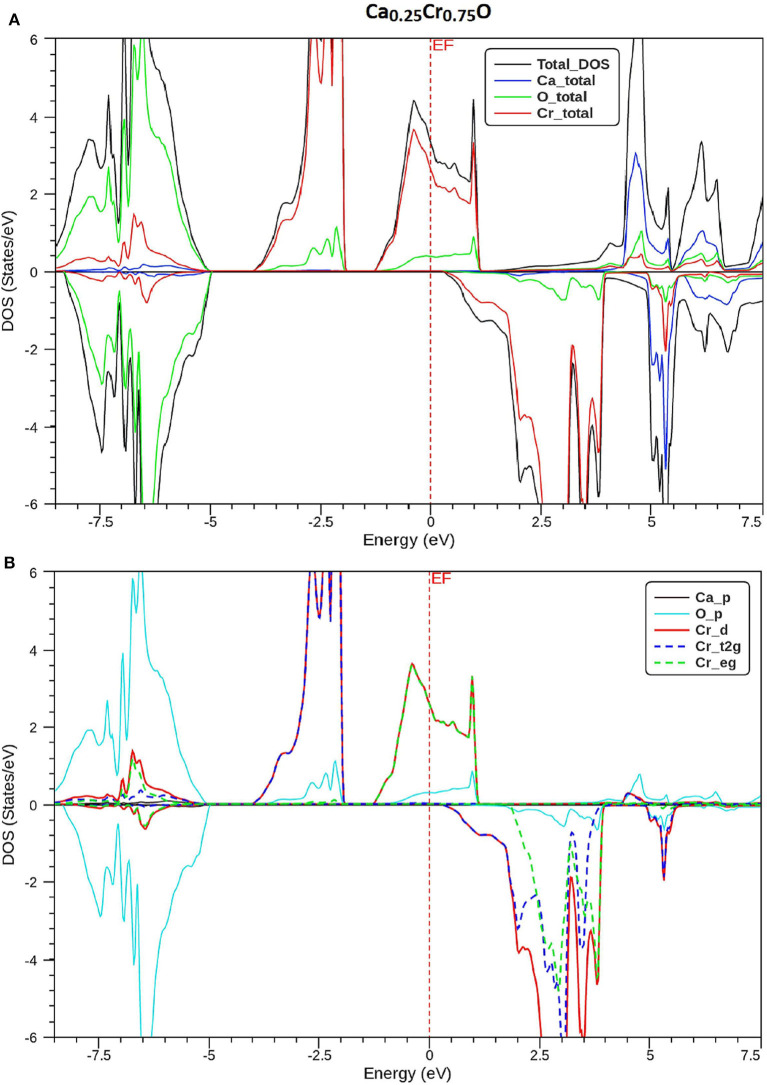
Spin-polarized total and partial densities of states (DOS) of Ca_0.25_Cr_0.75_O. The Fermi level is set to zero (*vertical dotted line*). **(A)** Total DOS of Ca, Cr, and O and **(B)** Partial DOS of p-Ca, p-O, d-Cr, t2g_d-Cr, and eg_d-Cr.

In order to understand the origin of half-metallic feature induced by the substituting effect of Cr, we have plotted the total and partial densities of states (DOS) of Cr, Ca, and O atoms of Ca_1-x_Cr_x_O compounds. Figures 8–10 show, respectively, the DOS of Ca_0.75_Cr_0.25_O, Ca_0.5_Cr_0.5_O, and Ca_0.25_Cr_0.75_O. We clearly see that all compounds are metallic for majority spin and semiconductors for minority spin, where their bonding, non-bonding, and anti-bonding states are distinguished from each other by the contributions of the DOS formed by different orbitals of each Ca, O, and Cr atoms.

The partial DOS of Figures 8–10B revealed that the 3d-Cr states are splitted into the low-lying *t*_2g_ and high-lying *e*_g_ states owing to the effect of octahedral crystal field created by neighboring oxygen (O) ions. For both minority and majority spins, the bonding states are mainly contributed by the p-O states and by the small p-Ca and 3d-Cr states in the ranges of −6.5 to −4, −7.5 to −4.5, and −8.25 to −5.25 eV, respectively, for Ca_0.75_Cr_0.25_O, Ca_0.5_Cr_0.5_O, and Ca_0.25_Cr_0.75_O. For the majority spins of Ca_1-x_Cr_x_O, the non-bonding states are principally dominated by the *t*_2g_-(3d-Cr) fully filled levels that hybridize largely with p-O orbitals, while the anti-bonding states are mostly contributed by the hybridization of the *e*_g_-(3d-Cr) levels and p-O states around *E*_F_. However, the upper part of valence bands of majority spins is originated from both *t*_2g_-(3d-Cr) non-bonding states and *e*_g_-(3d-Cr) anti-bonding states, which extend strongly in the gap owing to broadening of 3d-Cr levels around *E*_F_.

Furthermore, the upper part of valence bands of minority spins are formed by the bonding states originated from the p-O orbitals, which are centered at −5.25, −6, and −6.75 eV for Ca_0.75_Cr_0.25_O, Ca_0.5_Cr_0.5_O, and Ca_0.25_Cr_0.75_O, respectively. The bottom of conduction band is generated predominantly by the 3d-Cr empty anti-bonding states, which move to lower energies toward Fermi level from Ca_0.75_Cr_0.25_O, Ca_0.5_Cr_0.5_O, to Ca_0.25_Cr_0.75_O with increasing Cr concentration. Therefore, the distinguish half-metallic ferromagnetic and half-metallic gaps are created around *E*_F_ in minority spins for Ca_0.75_Cr_0.25_O, Ca_0.5_Cr_0.5_O, and Ca_0.25_Cr_0.75_O materials.

The spin-polarized material is known by its spin polarization of electronic structures, which is an important factor for characterizing a half-metal. The polarization (*P*) of half-metal compound results from the different contributions of numbers of DOS around *E*_F_. It is determined from the following expression (Soulen et al., 1998; Wang et al., 2017):

(2) $$\begin{array}{l} P=\frac{\left|N↑\left(E_{F}\right)-N↓\left(E_{F}\right)\right|}{\left|N↑\left(E_{F}\right)+N↓\left(E_{F}\right)\right|} \end{array}$$

where *N* ↑ (*E*_*F*_) and *N* ↓ (*E*_*F*_) indicate, respectively, the DOS of majority and minority spins at *E*_F_. From Figures 8–10A, the density of states *N* ↓ (*E*_*F*_) equals zero because the DOS do not cross *E*_F_, leading to a spin polarization of 100%. Therefore, the Ca_1-x_Cr_x_O compounds appear to be suitable candidates for possible spin-injection in spintronics applications.

### Magnetic Properties

#### Magnetic Moments

The spin-polarized electronic structures show an asymmetric character, indicating that the Ca_1-x_Cr_x_O compounds are magnetic in nature. The magnetism is induced by the magnetic spins arising from the localized 3d-Cr majority-spin states around *E*_F_. The Cr impurity contributes two electrons to host carriers of valence bands, resulting in Cr^+2^ ion. Thus, the 3d-Cr states become partially filled with four electrons. Therefore, the magnetism is originated from the four itinerant electrons of Cr^+2^ ions in Ca_1-x_Cr_x_O doping materials.

From the DOS of majority spins, one can see that the non-bonding states enclose three low-lying *t*_2g_ levels, which are completely occupied by three electrons. In contrast, the two high-lying *e*_g_ states have an anti-bonding nature, which are partially occupied by one electron because they are crossed by Fermi level in the middle. According to the Hund's rule, the 3d-Cr states generate a total magnetic moment of 4 μ_B_ per Cr ion. Consequently, the Ca_0.75_Cr_0.25_O, Ca_0.5_Cr_0.5_O, and Ca_0.25_Cr_0.75_O compounds have total magnetic moments of 4, 8, and 12 μ_B_, respectively, for one, two, and three Cr doping impurities.

The calculated total and partial magnetic moments of Ca, Cr, and O atoms and in the interstitial sites for Ca_0.75_Cr_0.25_O, Ca_0.5_Cr_0.5_O, and Ca_0.25_Cr_0.75_O are given in Table 3. The total magnetic moment per Cr atom is an integer number of 4 μ_B_. It is a typical feature of half-metallic materials (Wang et al., 2017; Zhang et al., 2017). All compounds have negative partial magnetic moments for O atoms, revealing that carriers of host valence bands enclosing p-O states interact anti-ferromagnetically with 3d-Cr states. Besides, the positive partial magnetic moments of Cr and Ca atoms describe the ferromagnetic interaction between Cr and Ca magnetic spins.

**Table 3:** Computed total and partial magnetic moments of the relevant Cr, Ca, and O atoms and in the interstitial sites (in Bohr magneton μ_B_) for Ca_1-x_Cr_x_O at concentrations *x* = 0.25, 0.5, and 0.75.

**Material**	**Total**	**Cr**	**Ca**	**O**	**Interstitial**
Ca_0.75_Cr_0.25_O	4	3.652	0.009	−0.075	0.414
Ca_0.5_Cr_0.5_O	8	7.203	0.011	−0.049	0.835
Ca_0.25_Cr_0.75_O	12	10.541	0.009	−0.059	1.391

#### Exchange Splitting Mechanisms

The ferromagnetism is created from the localized electrons of *t*_2g_ and *e*_2g_ of 3d-Cr states mediated by holes, where the empty holes of *e*_g_ states occur at the top of valence band and generate an acceptor hole type carrier hybridizing with carriers of host valence band (Doumi et al., 2013). The degenerate 3d-Cr into *t*_2g_ and *e*_2g_ is due to crystal field generated from the electrostatic environment of surrounding oxygen (O) ions, where the 3d-Cr majority-spin levels contain four unpaired electrons (Noor et al., 2018). According to the Hund's rule, this kind of process mediates magnetism in Ca_1-x_Cr_x_O materials (Yaqoob et al., 2019). It is understood that both exchange splitting of 3d-Cr states and crystal field take part in the contribution of ferromagnetism in Ca_1-x_Cr_x_O doping materials.

The magnitude of crystal field energy (Δ*E*_*CF*_) is defined as a difference between *E*_eg_ and *E*_t2g_ energies of *e*_g_ and *t*_2g_ states (Δ*E*_*CF*_ = *E*_*eg*_ − *E*_*t*2*g*_). The direct exchange splitting energy Δ_*x*_(*d*) = *d↓* − *d↑* is determined from the separation between the empty d-Cr (*d↓*) minority-spin and occupied d-Cr (*d↑*) majority-spin peaks. Another factor is used to determine the magnitude of ferromagnetism such as the indirect $\Delta_{x}\left(pd\right)=E_{v}^{↓}-E_{v}^{↑}$ exchange splitting resulted from the energy difference between $E_{v}^{↓}$ and $E_{v}^{↑}$ valence band maximums of minority- and majority-spin channels, respectively.

The values of Δ*E*_*CF*_, Δ_*x*_(*d*), and Δ_*x*_(*pd*) exchange splitting are shown in Table 4. The obtained values of (Δ*E*_*CF*_ and Δ_*x*_(*d*)) are (1.71 and 4.98 eV), (2.97 and 4.90 eV), and (1.74 and 5.17 eV), revealing that the direct exchange splittings are more dominated compared to the crystal field splittings. Therefore, the ferromagnetism state is mainly favored by the contribution of direct exchange splitting mechanism than that of crystal field (Amin et al., 2018). Besides, the partial magnetic moment of Cr increases from 3.652 μ_B_ for Ca_0.75_Cr_0.25_O, 7.203 μ_B_ for Ca_0.5_Cr_0.5_O, to 10.541 μ_B_ for Ca_0.25_Cr_0.75_O with decreasing Δ_*x*_(*pd*), meaning that magnetic moment of Cr is directly related to the indirect exchange splitting. On the other hand, the attraction nature of spin-polarized electrons is measured from the indirect exchange splitting (Verma et al., 2011; Mahmood et al., 2016, 2018). The indirect exchange splittings Δ_*x*_(*pd*) have negative values because the valence band maximums of minority-spins are situated at lower energies with respect to the valence band maximums of majority-spins. This means that the minority-spins have more attractive (negative) potential than that of majority-spins, leading to perfectly localized magnetic states. Consequently, the ferromagnetism is preferred in the Ca_1-x_Cr_x_O compounds. In the DMS based on III–V and II–VI semiconductors doped with transition metals, when the d states of magnetic dopants are partially occupied (Sato and Katayama-Yoshida, 2002; Sato et al., 2003a,b), the stability of ferromagnetism is described by the double-exchange mechanism (Akai, 1998). According to this rule, the anti-bonding states of partially filled 3d-Cr atoms associated with double-exchange mechanism stabilize the ferromagnetic state configuration in Ca_1-x_Cr_x_O.

**Table 4:** Computed energies of crystal field Δ*E*_*CF*_, direct Δ_*x*_(*d*), and indirect Δ_*x*_(*pd*)exchange splittings for Ca_1-x_Cr_x_O at concentrations *x* = 0.25, 0.5, and 0.75.

**Material**	**ΔE_CF_ (eV)**	**Δ_x_(d) (eV)**	**Δ_x_(pd) (eV)**
Ca_0.75_Cr_0.25_O	1.71	4.98	−3.75
Ca_0.5_Cr_0.5_O	2.97	4.90	−4.25
Ca_0.25_Cr_0.75_O	1.74	5.17	−4.97

The magnitudes of valence and conduction band edge splitting are measured by the two sp-d exchange constants *N*_0_α and *N*_0_β. The *N*_0_α parameter determines the s-d exchange interaction between the s carriers of conduction bands and d-Cr levels, while the *N*_0_β describes the p-d exchange coupling between the p states of carriers of valence bands and d-Cr levels. We have used the mean field theory (Sanvito et al., 2001; Raebiger et al., 2004) to calculate the exchange constants of Ca_1-x_Cr_x_O compounds given by the following relations.

(3) $$\begin{array}{c} N_{0}\alpha =\frac{\Delta E_{c}}{x\langle s\rangle } \end{array}$$

(4) $$\begin{array}{c} N_{0}\beta =\frac{\Delta E_{v}}{x\langle s\rangle } \end{array}$$

The $\Delta E_{v}=E_{v}^{↓}-E_{v}^{↑}$ and $\Delta E_{c}=E_{c}^{↓}-E_{c}^{↑}$ difference energies correspond, respectively, to the valence band-edge and conduction band-edge spin-splittings at Γ high symmetry point. The 〈*s*〉 and *x* are the half total magnetic moment per Cr and the concentration of Cr, respectively (Sanvito et al., 2001). The calculated Δ*E*_*v*_, Δ*E*_*c*_, *N*_0_α, and *N*_0_β are summarized in Table 5. Yaqoob et al. (2019) have predicted that the negative values of *N*_0_α and *N*_0_β are due to the quantum confinement effect or structural symmetry changes that modify the coupling of different states. For Ca_1-x_Cr_x_O materials, the negative values of *N*_0_α and *N*_0_β describe the anti-ferromagnetic coupling between the valence and conduction bands and the 3d states of magnetic Cr atoms.

**Table 5:** Computed conduction band-edge Δ*E*_*c*_(eV) and valence band-edge Δ*E*_*v*_(eV) spin-splittings, and sp-d exchange constants *N*_0_α and *N*_0_β for Ca_1-x_Cr_x_O at concentrations *x* = 0.25, 0.5, and 0.75.

**Material**	**Δ*E*_c_ (eV)**	**Δ*E*_v_ (eV)**	***N*_0_α**	***N*_0_β**
Ca_0.75_Cr_0.25_O	−0.858	−3.030	−1.716	−6.060
Ca_0.5_Cr_0.5_O	−0.741	−3.643	−0.741	−3.643
Ca_0.25_Cr_0.75_O	−0.972	−4.431	−0.648	−2.954

## Conclusions

We have characterized the structural properties, the spin-polarized electronic structures, and ferromagnetic performance induced by the Cr impurities in Ca_1-x_Cr_x_O compounds at concentrations *x* = 0, 0.25, 0.5, and 0.75. The Ca_1-x_Cr_x_O doping materials are dynamically stable due to their positive phonon frequencies. The structural parameters and indirect gap of CaO are very significant with respect to the recent calculations found by GGA-WC and TB-mBJ. For Ca_1-x_Cr_x_O, the lattice constant decreases with increasing Cr concentration, leading to the increase in bulk modulus, where the sizes of the ionic radii of Ca and Cr atoms are the factors determining these changes. The Ca_1-x_Cr_x_O systems have a half-metallic ferromagnetic feature, where the ferromagnetism is favored by the large contribution of direct exchange splitting than that of the crystal field. The analysis of magnetic properties shows that both double-exchange and p-d exchange mechanisms participate to stabilize ferromagnetic state configuration. The half-metallic ferromagnets Ca_1-x_Cr_x_O compounds maintain a half-metal gap feature for all concentrations with spin polarization of 100%, making them suitable spin-injection candidates for possible exploration in semiconductors spintronics.

## Data Availability Statement

All datasets presented in this study are included in the article/supplementary material.

## Author Contributions

With the submission of this manuscript, all authors declare that: The manuscript is original. All authors of this research paper have contributed to this manuscript. All authors of this paper have read and approved the final version submitted. The contents of this manuscript have not been copyrighted or published previously. The contents of this manuscript are not now under consideration for publication elsewhere.

## Conflict of Interest

The authors declare that the research was conducted in the absence of any commercial or financial relationships that could be construed as a potential conflict of interest.
